# Population-oriented health promotion and disease prevention interventions in primary healthcare: a scoping review of reviews

**DOI:** 10.1186/s12875-026-03337-y

**Published:** 2026-04-22

**Authors:** Leif Eriksson, Karin Olsson, Henna Hasson, Anna Bergström, Veronica-Aurelia Costea, Emma Holm, Annika Bäck

**Affiliations:** 1 Center for Epidemiology and Community Medicine, Region Stockholm, Stockholm, Sweden; 2 Department of Learning, PROCOME Research Group, Medical Management Centre, Informatics, Management and Ethics, Karolinska Institutet, Stockholm, Sweden

**Keywords:** Primary healthcare, Health promotion, Disease prevention, Scoping review, Population-oriented

## Abstract

**Background:**

Primary healthcare is located close to the population and is key to providing health promotion and disease prevention interventions, which are important strategies to achieve universal health coverage. Population-oriented interventions provided by primary healthcare have been reported as effective, but research has not presented a global overview of their characteristics yet. Therefore, the purpose of this scoping review of reviews is to map population-oriented health promotion and disease prevention interventions within primary healthcare.

**Methods:**

Four databases (MEDLINE, Web of Science, PsycInfo, and CINAHL) were searched for various types of reviews (systematic reviews, scoping reviews, realist reviews, rapid reviews, realist synthesis, narrative reviews, critical reviews, and other types of reviews if they provided enough information about a systematic approach) published in English from inception to 2022. Reviews were included and data extracted if their results sections reported on articles meeting our inclusion criteria about participants, concept, context, and type of evidence source. The analysis entailed summarising extracted data for some items while items with more extensive information underwent a qualitative content analysis.

**Results:**

Of 9,254 screened reviews, 310 reviews containing 2,426 articles meeting the predetermined inclusion criteria were included. Interventions were provided globally, but most were provided in the Region of Americas (*n* = 974) followed by the European Region (*n* = 596). The interventions targeted different areas, where the most common areas were: Cardiovascular disease/diabetes; Perinatal and child health; Sexual and reproductive health; Cancer; and Mental health. The most common intervention components were Counselling/Education followed by Screening, and Referral/Invitation. The most common actor was a General Practitioner, the most common setting was a Primary Care Clinic, and the largest target group was Adults. However, there were differences regarding intervention components, actors, target groups and setting across the target areas.

**Conclusions:**

This scoping review provides a comprehensive overview of population-oriented preventive and health promoting interventions that have been provided in primary healthcare. Most health promotion and disease prevention interventions are delivered within clinical settings, often on an individual basis. The results can be used by various types of stakeholders for further development of interventions, planning of implementations, and as an indication for future research focus.

**Supplementary Information:**

The online version contains supplementary material available at 10.1186/s12875-026-03337-y.

## Background

Primary healthcare is essential in achieving the Sustainable Development Goals (SDGs) overall, particularly Goal 3.8 which aims for universal health coverage by providing healthcare on the continuum of care, from health promotion and preventive activities targeting entire populations to treatment and palliative care targeting individual patients [1]. The 2018 Astana Declaration highlights three main functions of primary healthcare. First, it should provide a wide range of healthcare services throughout the lifespan. Secondly, primary healthcare should address broad determinants of health through multisectoral action, and lastly, it should empower the population to optimise their health through, for instance, self-care [1]. The concept *primary healthcare,* as opposed to *primary care*, encompasses services consisting of both individual-level care and population-focused public health activities. It rests on core principles, such as universal access to healthcare, a commitment to health equity, and intersectoral collaboration [2, 3]. Primary care is defined more narrowly as consisting of the provision of integrated, accessible health care services by clinicians who are accountable for addressing a large majority of personal health care needs, developing a sustained partnership with patients, and practising in the context of family and community [4]. This review focuses on the broader term primary healthcare, which also includes primary care.

In many countries, primary healthcare has been a focal point in clinical guidelines concerning health promotion and preventive services [5–7]. This is in line with the World Health Organization’s (2016) global strategy on integrated person-centred health services, which highlights the need to adopt a model of care that puts emphasis on promotion and prevention [8]. Primary healthcare staff are well positioned to work with health promotion and prevention as they interact with patients continuously and understand the local context. Further, many patients have risk factors for chronic diseases that are amenable to prevention within primary healthcare [9, 10]. However, challenges to provide preventive services in primary care clinics are many and can be found on multiple levels: individual (e.g., lack of provider time and lack of knowledge among patients), organisational (e.g., lack of patient information, lack of structural and financial accessibility of preventive services), and health system levels (e.g., socio-cultural factors and unclear roles of health professionals in prevention) [10]. In addition, primary healthcare is also needed to act as a link between patients and community resources to increase the capacity for prevention and health promotion on a population level [9], as well as to provide population-based interventions. Aside from providing quality care, primary healthcare may address further social determinants of health by partnering with the community to improve population health through creating healthy environments, advocating for social change, and engaging in community health planning [11].

Some evidence indicates that a population-based approach targeting, for example, weight reduction, improving nutrition, and increasing physical activity could be more effective than usual individual care [12]. Population-based primary prevention interventions in primary healthcare have further been found to be effective in reducing cardiovascular risk factors [13]. Despite this, population-based approaches are still underutilised in primary healthcare. A scoping review on health promotion and disease prevention in UK primary care concluded that such interventions were provided but that the majority targeted the individual [14]. Other studies focusing on public health nurses have also found a predominance of health promotion and prevention work on an individual level, with population-based approaches rarely being utilised, despite governmental guidelines emphasising a more population-based approach to health promotion and prevention [15, 16]. This indicates a lack of population-based health promotion and prevention interventions within primary healthcare. Commonly quoted reasons for this include financial as well as cultural factors, as the role of primary healthcare is vague regarding the possibility of using population-based approaches. Further, there is a need for increased knowledge among staff on how to employ a population-based approach [10, 15, 16].

The purpose of this scoping review of reviews is to map population-oriented health promotion and disease prevention interventions within primary healthcare to provide knowledge on the following research questions:

- What kind of interventions have been delivered?
- Which actors have been involved in delivering these interventions?
- In which settings have these interventions been delivered?
- What population groups have been targeted?
- How have the population groups been recruited/identified?

## Methods

The scoping review methodology is deemed appropriate to obtain an overview of a broad topic [17, 18]. Results from a scoping review can inform future research efforts [19]. This scoping review followed the guidance provided by Peters et al. 2020 [20] and their updated guidance [21]. Due to the broad search area, the search was focused on reviews followed by identification of original articles in the included reviews, i.e. this is a scoping review of reviews. The methods section provides details about 1) Inclusion criteria, 2) Search strategy, 3) Evidence screening and selection, 4) Data extraction, 5) Data analysis, and 6) Presentation of results [20, 21]. Reporting adheres to the PRISMA-ScR checklist [22] (Additional file 1). The study was registered on the Open Science Framework (10.17605/OSF.IO/7MCR9).

### Inclusion criteria

#### Participants

We included articles that describe interventions provided to:

- Target groups in different stages of life without a specific diagnosis, (e.g., healthy children, pregnant women, adults, and elderly);
- Target groups with a diagnosis, if the intervention focused on another problem than the diagnosis, or if the intervention aimed to prevent the spread of a disease (e.g. Human Immunodeficiency Virus);
- Target groups with lifestyle-related risk factors (e.g. overweight, and harmful use of alcohol or tobacco); and
- Caregivers for family members.

We excluded articles that described interventions targeting healthcare staff and interventions targeting groups with a diagnosis, and thus not preventive in nature (e.g., treatment for cancer).

#### Concept

We included articles where:

- The intervention(s) aimed to prevent disease (primary and secondary prevention) or promote health;
- Intervention(s) aimed at strengthening the population's ability to assist others in emergencies (e.g. handling cardiac arrest, fainting or bleeding in others); and
- A description of the intervention component(s) was available.

We followed the World Health Organization’s definitions of disease prevention and health promotion [23]:

“*Disease prevention*, understood as specific, population-based, and individual-based interventions for primary and secondary prevention, aiming to minimise the burden of diseases and associated risk factors. Primary prevention refers to actions aimed at avoiding the manifestation of a disease” … ”Secondary prevention deals with early detection when this improves the chances for positive health outcomes.”

“*Health promotion* is the process of empowering people to increase control over their health and its determinants through health literacy efforts and multisectoral action to increase healthy behaviours. This process includes activities for the community-at-large or populations at increased risk of negative health outcomes.”

Although the World Health Organization acknowledges substantial overlap in the goals and functions of these activities, it also highlights that they differ in their primary focus: disease prevention is centred within the healthcare system, while health promotion relies on intersectoral collaboration and targets the broader determinants of health [23].

We excluded articles describing interventions for treating or managing disease (e.g., introduction or withdrawal of medication and screening of patients seeking care with specific symptoms), tertiary prevention (reducing the progress or complications of established disease), and health promotion aimed at individuals or groups with a diagnosis or controlled chronic disease/symptoms. We also excluded articles that focused on assessing barriers and facilitators to implementing an intervention, attitudes of staff implementing an intervention, and cost analysis of an intervention.

#### Context

Articles were included:

- When a primary care clinic was involved, i.e. either as the setting where the intervention was provided or connecting the target group with actors in another setting, e.g., by referral or recommendation;
- If the care provider in the intervention had the equivalent role to primary care clinics, as the first point of contact for care, e.g., dental care, pharmacy services, student health services, midwifery services or child health care centres. Hence, such a provider is considered part of the broader primary healthcare; and
- If the setting was not specifically mentioned, but the actor providing the intervention was judged to belong to one of the primary healthcare settings, e.g. a general practitioner (GP) or community health worker (CHW).

Articles were excluded when the actor providing the intervention was not from primary healthcare or the first point of contact for care, e.g., an actor providing inpatient care at hospitals, specialist care, or hospital emergency care. We also excluded articles describing interventions delivered in settings inaccessible to the general public, such as prisons or workplaces (occupational health services).

#### Types of evidence sources

We included articles that were:

- Published in English; and
- Peer-reviewed reviews of these types: systematic review, scoping review, realist review, rapid review, realist synthesis, narrative review, and critical review; and
- Other types of reviews, if they provided enough information about a systematic approach (i.e. available inclusion/exclusion criteria, search terms, databases searched, and a presented PRISMA flow chart).

We excluded articles that were not published in English; non-empirical studies and study protocols, and reviews not providing information about search terms, databases accessed, inclusion/exclusion criteria and a PRISMA flow chart. To avoid duplicates among articles we also excluded older versions of a review, e.g., in case of Cochrane reviews with multiple versions, only the most recent update was included.

### Search strategy

A search strategy was developed in collaboration with an information specialist from Karolinska Institutet University Library, Sweden, who searched four electronic databases: MEDLINE (OVID), Web of Science (Clarivate), PsycInfo (EBSCO), and CINAHL. The search process, including the refinement of search terms to identify articles that met the inclusion criteria, lasted from May to July 2022. All search strategies performed in the four databases are available in Additional file 2. De-duplication was performed using the method described by Bramer et al. (2016) [24].

### Evidence screening and selection

Title and abstract screening of the articles was conducted in Rayyan [25]. Each article was screened independently by two reviewers using the inclusion criteria described above. Four authors (LE, KO, EH, ABä) were involved in this step. Arising conflicts were discussed and solved between the two reviewers, and with a third reviewer, if necessary. If consensus was not reached, the abstract was included for full-text screening.

The articles judged as relevant for full-text screening and available in full-text format were imported to the software Covidence [26]. The full-text screening of each article was performed by two reviewers independently. The review was included if having at least one article (based on the available information in the Results section of the review) that met the inclusion critera for participants, concept, context, language, and type of publication. Four authors (LE, KO, EH, ABä) were involved in this step. Conflicts were solved between the two reviewers, and if there were disagreement they were brought to the full author group at weekly meetings and discussed until consensus was reached.

### Data extraction

A data extraction template was developed, tested, and revised prior to extraction. The final extraction items are presented in Table 1 and defined in Additional file 3. Both data about the characteristics of the reviews and from the Result sections of the reviews were extracted. The latter involved extracting information about the characteristics of the articles included in the results of the reviews and meeting our inclusion criteria. No original articles were retrieved, instead only the information available in the review was used for analysis. Two reviewers separately extracted data from each review. Four authors (LE, KO, EH, ABä) were involved in this step.

**Table 1:** Overview of items for data charting

**Characteristics of the review**
Study ID (Last name first author and Year of publication)
Title
Aim of review
Type of review
Databases sourced and searched in review
Time range of database search in review
Target area
Number of articles from the review relevant to the target health area studies from review relevant to target area
**Characteristics of articles in result section of review**
First author
Publication year
Study design
Country
Target group
Description of intervention
Actors involved
Setting
How target group was offered intervention
Sample size

### Data analysis

Extracted data were exported to Excel to be stored and analysed. The items target area, intervention component, target group, actor, setting, and descriptions of how the target group was offered the intervention component(s) were analysed using inductive qualitative content analysis [27]. This entailed reading and coding each extract. Thereafter, codes were grouped into categories. Definitions of items and identified categories are available in Additional file 3. The data synthesis was performed by three of the authors (LE, KO, ABä). All authors discussed the synthesis and how to present the results.

It was anticipated that articles would appear in more than one review and it was discussed in the author group if duplicates should be removed or not. It was decided not to remove duplicates as this would force us to remove them from specific reviews, potentially resulting in excluding entire reviews. Further, we noted that individual articles were included in reviews aiming to study different things and thus the information available varied in each review. No logic resoning could be found in the author group about how to remove duplicates without implications. Hence, the duplicates are represented in the data but the estimated proportion of duplicates (number of duplicate articles/total number of articles × 100) are provided in the Results section for each target area. We estimated the number of duplicate original articles included in the reviews based on available information in each review by using the following steps: 1) Identify articles with the same author, 2) Compare available information between the articles, i.e. sample, year, design, country, etc., and 3) Count as duplicate if comparisons matched and no contradicting information was detected. To reduce the number of duplicates, only the last updated reviews were included when reviews had been updated more than once.

### Presentation of results

Results are presented narratively in figures, tables, and a box. The Results section first provides an overview of identified reviews and the screening process in a PRISMA diagram. Thereafter, overall results are presented in response to the aim and the research questions, followed by a section where data is presented for the identified target areas. All data for the reviews and accompanying articles are available in Additional file 4.

## Results

**Fig. 1:**
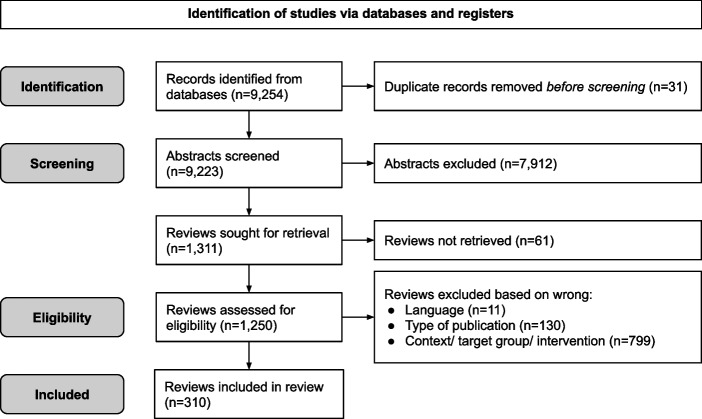
Prisma flow diagram

The search generated 9,254 records. After removal of duplicates (i.e., same review articles) (*n* = 31), 9,223 records remained, which underwent screening of abstracts. Full-text screening was thereafter performed for 1,250 eligible and available reviews. In total, 310 reviews [28–337] remained in the final sample, i.e. the reviews having any articles in their Results section that met our inclusion criteria. The PRISMA flow diagram presents the process of selecting the included articles (see Fig. 1).

### Review characteristics

**Fig. 2:**
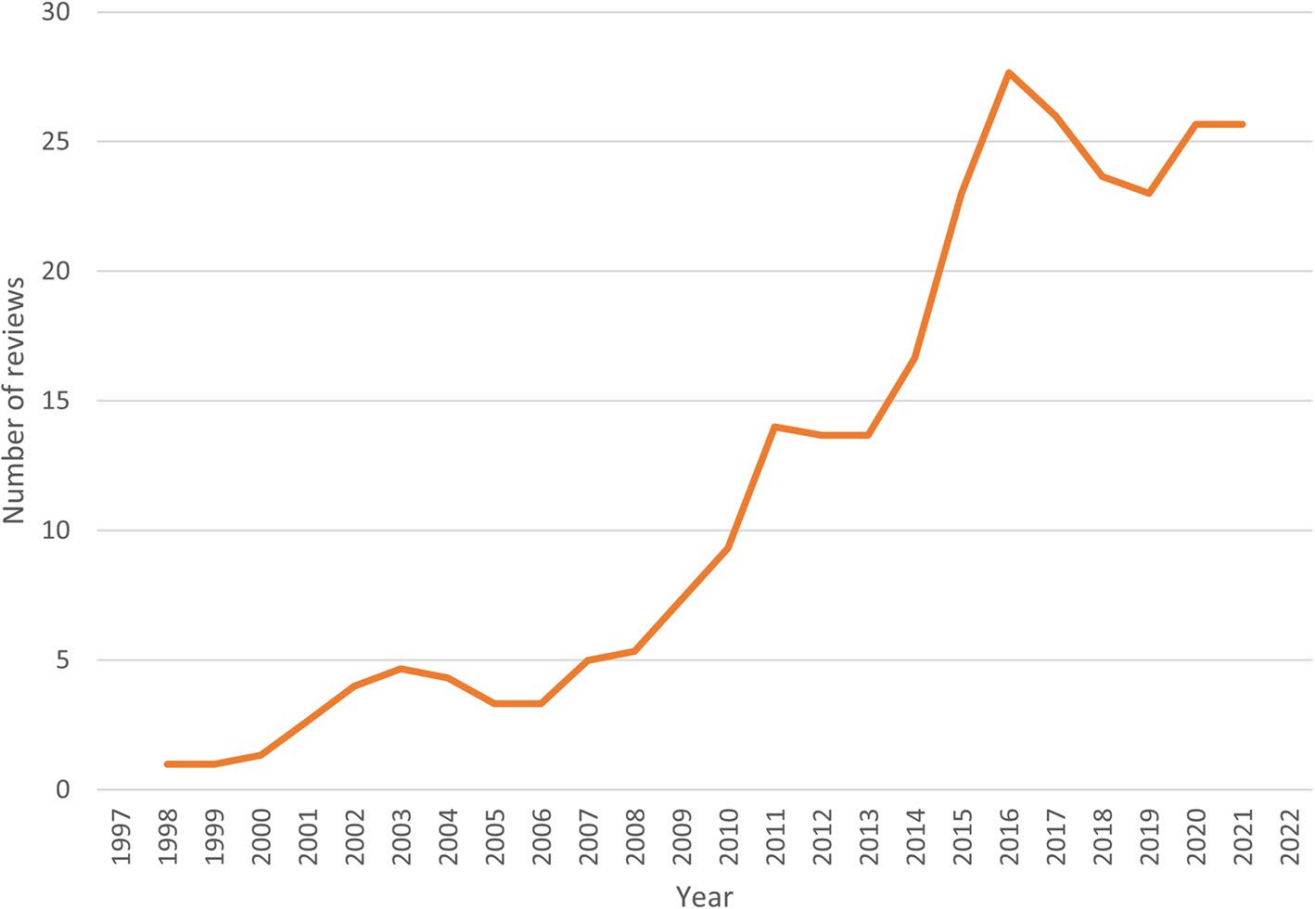
Number of reviews by year expressed as three-year moving averages

The 310 included reviews were published between 1997 and 2022. Up until 2016, there was a steady increase in the number of reviews, after which the trend seems to have declined (see Fig. 2). As the search was performed during the first part of 2022, this year should be considered incomplete. While the study includes various types of reviews, systematic reviews constituted the majority (*n* = 264) while other reviews were less common, for example scoping reviews (*n* = 22); unspecified, but systematic reviews (*n* = 9); and integrative reviews (*n* = 7). In the Results sections of the included reviews, a total of 2,426 original articles met the inclusion criteria for the current study (the number of included articles from each review varied between 1 and 50).

### Overview of interventions, target groups, actors, and settings

**Fig. 3:**
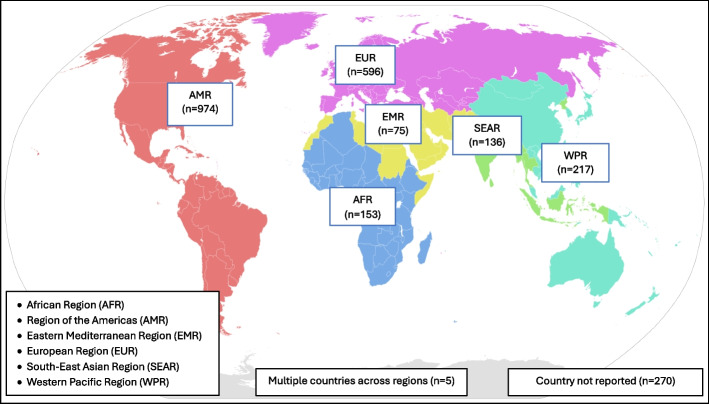
Distribution of articles from reviews according to World Health Organization regions

The interventions described in the articles were provided across a variety of geographic locations (see Fig. 3). Among the six regions of the World Health Organization [338], most interventions were provided in the Region of the Americas, where USA was the country with most articles (n = 870). The European Region also contributed to many of the articles, but with a larger number of countries contributing to the total number of interventions. All available data extracted for each review and associated articles is presented in Additional file 4.

The interventions often encompassed several intervention components. A total of 14 intervention components were identified (see Table 2). Some components significantly vary in intensity and depth. For instance, *Counselling/Education* ranges from providing informational material to delivering a comprehensive counselling program over several weeks. Also examples of programs lasting for more than two years existed. In contrast, other components, such as *Vaccination*, are more narrowly defined and more uniform in scope.

**Table 2:** Intervention components

Types of intervention components provided (n)	Definitions
Counselling/Education (1968)	Counselling, e.g., brief advice, motivational interviewing, and therapy. Education, e.g., group/individual educational lectures/materials. Information, e.g., flyers, brochures, or posters with educational messages
Screening (658)	Screening/testing for early disease detection; assessment of health status for potential follow-up/referral
Referral/Invitation (284)	Referral/invitation/linkage to a health-promoting activity or examination (e.g., a formal invitation or direct referral from a doctor, but also a less direct linkage, such as provision of community resource lists)
Reminder (176)	Reminder for screening/health check or other health-promoting activity
Incentives (156)	Some form of incentive or gift to increase motivation to engage in a health-promoting activity, e.g. a voucher, an oral health kit or pedometers
Pharmacology/Nutrition (107)	Pharmacological or nutritional support e.g. distribution of nicotine replacement therapy, nutritional supplements, contraceptive pills, etc
Practical Support (89)	Practical support aimed at overcoming barriers to health-promoting interventions, e.g., transportation to/from screening or changes in one’s local environment/home to promote healthy behaviours or reduce risk of accidents
Physical Activity (65)	Physical activity e.g., walking groups or exercise programs
Skills Training (51)	Teaching practical skills to prevent disease and/or improve health, e.g., learning to perform self-breast exams for breast cancer, to cook healthy recipes or mental coping skills to avoid psychological distress
Social Activity (26)	Social/cultural activities to enhance a health message or counteract social isolation, e.g., communal dinners, theatres, or exhibitions
Media (24)	Use of media or marketing to disseminate health-related messages to the target group
Inspiration/Support (18)	Intervention using an inspirational person to influence or motivate the target group towards better health, or a person with personal experience of the targeted health situation/condition providing support
Mobilization (18)	Mobilizing community groups in different ways to encourage desired health promoting or disease prevention activities, e.g., screening
Vaccination (17)	Providing vaccination

*n* number of articles

Twelve different types of actors were identified (see Table 3). Some of these, such as *GP*, *Nurse* and *Midwife* were easily labelled during the analysis. Other actor groups are based on functions (see Additional file 2). For instance, the category *CHW* contains not only the actors specifically titled as such, but also those with different titles performing the same function.

**Table 3:** Different types of actors, target groups, and settings

Types of actors involved (n)	Population groups targeted (n)	Settings for intervention (n)
• GP (575)	• Adults (843)	• Primary care clinic (1,197)
• CHW (474)	• Risk Group (618)	• Home (423)
• Nurse (417)	• Pregnant/Postpartum (528)	• Perinatal/Child healthcare clinic (303)
• Health Worker (127)	• Women (368)	• Community (154)
• Counsellor (110)	• Older Adults (326)	• Sexual Healthcare clinic (93)
• Other Professions (76)	• Caregivers (274)	• Pharmacy (78)
• Pharmacy Staff (66)	• Children > 5 (265)	• Dental Clinic (57)
• Project Staff (58)	• Minority Group (204)	• School (36)
• Midwife (50)	• General Population (167)	• Other (45)
• Specialist Medical Doctor (49)	• Children ≤ 5 (152)	
• Supporting Position (45)	• Men (78)	
• Dental Staff (40)	• Migrants (34)	

*n * number of articles, *GP* General Practitioner, *CHW* Community Health Worker

When describing the target groups, we found 12 different categories that were either presented individually (e.g. *Children* > *5* or *Adults*) or in combination (e.g. *Older adults* + *Men*). Eight types of settings were found, i.e., different types of *Clinics, Home, School, Pharmacy* and *Community*. The group *Other* consists of less common settings, such as Hospital, Health Maintenance Organization, and Immunisation clinic. The methods used to invite or identify target groups were only reported in 648 articles and included *Recruitment at a meeting* (e.g. in a clinic, at home or another setting), *Letter/phone call*, *Identification in e.g., registers or through surveys*, and *Advertisement/media*. See Additional file 3 for more details.

### Overview of target areas

The interventions were grouped into 11 different target areas (see Table 4). The largest target area was *Cardiovascular disease/Diabetes*, covering different types of interventions aimed at promoting healthy lifestyle habits to prevent Cardiovascular disease and Diabetes. The remaining ten areas cover a wide range of health issues, from mental health promotion interventions to cancer prevention interventions.

**Table 4:** Overview of interventions’ target areas, content and number of articles

Target area	Intervention content	Number of articles
1. Cardiovascular disease/Diabetes	Diet, alcohol, physical activity, smoking, weight	1,044
2. Perinatal and child health	Women's and children’s health throughout pregnancy and before/during/after childbirth. Children’s health from birth to age 5. Parenthood, child development	377
3. Sexual and reproductive health	Contraceptives, reproduction, and family planning. Prevention of teenage pregnancies and sexually transmitted diseases (STDs)	239
4. Cancer	Different methods of detection and prevention of cancer, as well as encouraging individuals to take action	235
5. Mental health	Prevention related to loneliness, suicide, depression, etc	165
6. Communicable diseases	Prevention of communicable diseases such as Hepatitis, Malaria, Tuberculosis	99
7. Injuries, accidents and violence	Prevention of injuries/falls as well as domestic violence	98
8. Oral health	All aspects of oral health	62
9. Drugs and other substances	Drugs and other substances	25
10. Conditions mostly affecting elderly	Related to frailty, osteoporosis, visual impairment, etc	24
11. Other	Includes organ donation, passive smoking and needs assessment	58
**TOTAL**		2,426

In Tables 5, 6, 7, 8, 9, 10, 11, 12, 13, 14 and 15, the results from the 11 target areas are presented. The tables show, for each target area, the number of reviews and articles, types of intervention components, actors, target groups, settings, and sample sizes.

**Table 5:** Target area cardiovascular disease/Diabetes

**Reviews (N)**	**Included articles (n)**	**Intervention component/s (n)**	**Actors involved (n)**	**Target group/s (n)**	**Setting where intervention was provided (n)**	**Sample size (n)**
117	1,044*	Number of intervention components: • One (695) • Multiple (349) Types of intervention components: • Counselling/Education (947) • Screening (225) • Referral/Invitation (76) • Pharmacology/Nutrition (55) • Incentive (52) • Physical activity (49) • Reminder (15) • Media (8) • Skills training (8) • Motivator (6) • Practical support (6) • Social activity (5) • Mobilisation (2)	Number of actors: • One (588) • Multiple (199) • Not reported (257) Types of actors: • GP (367) • Nurse (200) • CHW (117) • Health worker (64) • Counsellor (53) • Pharmacy staff (46) • Dental staff (29) • Other professions (28) • Project staff (26) • Specialist MD (14) • Midwife (13) • Supporting position (10) • Other (53)	Number of target groups: • One (927) • Multiple (117) Types of target groups: • Adults (564) • Risk group (483) • Elderly (150) • Pregnant/Postpartum (116) • Women (90) • Children > 5 (65) • Men (56) • General population (52) • Caregivers (48) • Minority group (46) • Children ≤ 5 (13) • Migrant (6)	Number of settings: • One (859) • Multiple settings (56) • Not reported (138) Types of settings: • Primary Care Clinic (630) • Home (114) • Perinatal/Child Healthcare Clinic (68) • Pharmacy (48) • Dental Clinic (48) • Community (32) • Sexual Healthcare Clinic (7) • School (3) • Other (15)	• < 100 (110) • 100–999 (634) • 1000–10,000 (209) • > 10,000 (23) • Not reported (68)

*CHW* Community Health Worker, *GP* General Practitioner, *MD* Medical Doctor, *N* Number of reviews, *n* Number of articles; *20–25% duplicates

**Table 6:** Target area perinatal and child health

Reviews (N)	Included articles (n)	Intervention component/s (n)	Actors involved (n)	Target group/s (n)	Setting where intervention was provided (n)	Sample size (n)
61	377*	Number of intervention components: • One (231) • Multiple (147) Types of intervention components: • Counselling/Education (311) • Screening (68) • Referral/Invitation (48) • Reminder (40) • Incentive (33) • Pharmacology/Nutrition (22) • Skills Training (18) • Practical Support (14) • Vaccination (8) • Mobilization (6) • Motivator (5) • Physical Activity (3) • Social Activity (2) • Media (1)	Number of actors: • One (206) • Multiple (68) • Not reported (104) Types of actors: • CHW (139) • Nurse (47) • Health worker (22) • GP (21) • Supporting Position (21) • Other Professions (21) • Midwife (17) • Specialist MD (13) • Counsellor (5) • Project staff (4) • Other (41)	Number of target groups: • One (289) • Multiple (89) Types of target groups: • Pregnant/Postpartum (245) • Caregivers (102) • Children** ≤ **5 (81) • Women (49) • Risk Group (41) • Children > 5 (22) • Minority Group (19) • Adults (13) • Migrants (8) • Men (4) • General Population (1)	Number of settings: • One (293) • Multiple settings (52) • Not reported (33) Types of settings: • Home (121) • Primary Care Clinic (113) • Perinatal/Child Healthcare Clinic (112) • Community (38) • School (10) • Other (9)	• < 100 (37) • 100–999 (142) • 1000–10,000 (73) • > 10,000 (26) • Not reported (99)

*CHW* Community Health Worker, *GP* General Practitioner, *MD* Medical Doctor, *N* Number of reviews, *n* Number of articles; *5–15% duplicates

**Table 7:** Target area sexual and reproductive health

Reviews (N)	Included articles (n)	Intervention component/s (n)	Actors involved (n)	Target group/s (n)	Setting where intervention was provided (n)	Sample size (n)
42	239*	Number of intervention components: • One (130) • Multiple (109) Types of interventions: • Counselling/Education (201) • Screening (84) • Referral/Invitation (18) • Incentive (18) • Reminder (12) • Skills training (14) • Pharmacology/Nutrition (10) • Social activity (6) • Practical support (6) • Mobilisation (2) • Media (2) • Vaccination (1)	Number of actors: • One (98) • Multiple (19) • Not reported (122) Types of actors: • CHW (33) • Counsellor (29) • GP (21) • Nurse (21) • Pharmacy staff (9) • Midwife (7) • Health worker (6) • Supporting position (5) • Other professions (2) • Specialist MD (2) • Project staff (1) • Other (9)	Number of target groups: • One (190) • Multiple (49) Types of target groups: • Children > 5 (97) • Adults (91) • Women (63) • Pregnant/Postpartum (62) • Minority (25) • General population (25) • Caregivers (14) • Risk group (13) • Men (12) • Children ≤ 5 (2) • Migrants (2)	Number of settings: • One (205) • Multiple (21) • Not reported (13) Types of settings: • Sexual Healthcare Clinic (80) • Primary Care Clinic (69) • Perinatal/Child Healthcare Clinic (41) • Home (22) • School (16) • Community (13) • Pharmacy (11) • Other (2)	• < 100 (24) • 100–999 (99) • 1000–10,000 (60) • > 10,000 (20) • Not reported (36)

*CHW* Community Health Worker, *GP* General Practitioner, *MD* Medical Doctor, *N* Number of reviews, *n* Number of articles, *5–15% duplicates

**Table 8:** Target area cancer

**Reviews (N)**	**Included articles (n)**	**Intervention component/s (n)**	**Actors involved (n)**	**Target group/s (n)**	**Setting where intervention was provided (n)**	**Sample size (n)**
25	235*	Number of intervention components: • One (101) • Several (134) Types of intervention components: • Counselling/Education (176) • Reminder (82) • Screening (51) • Referral/Invitation (50) • Practical support (44) • Incentive (13) • Media (7) • Skills training (6) • Motivator (4) • Mobilisation (3) • Social activity (2) • Physical activity (1)	Number of actors: • One (163) • Multiple (28) • Not reported (44) Types of actors: • CHW (91) • GP (80) • Project staff (11) • Nurse (10) • Health worker (4) • Other professions (4) • Dental staff (4) • Specialist MD (3) • Counsellor (3) • Pharmacy staff (3) • Midwife (1) • Supporting position (1) • Other (12)	Number of target groups: • One (183) • Multiple (52) Types of target groups: • Adults (134) • Women (113) • Minority group (84) • Older adults (50) • General Population (40) • Risk group (36) • Migrants (4) • Children > 5 (3) • Men (2) • Caregivers (1) • Children ≤ 5 (1)	Number of settings: • One (141) • Multiple (26) • Not reported (68) Types of settings: • Primary Care Clinic (102) • Home (40) • Community (33) • Pharmacy (12) • Perinatal/Child Healthcare Clinic (2) • Sexual Healthcare Clinic (1) • Dental Clinic (1) • Other (5)	• < 100 (10) • 100–999 (99) • 1000–10,000 (52) • > 10,000 (16) • Not reported (58)

*CHW* Community Health Worker, *GP* General Practitioner, *MD* Medical Doctor, *N* Number of reviews, *n* Number of articles, *5–15% duplicates

**Table 9:** Target area mental health

**Reviews (N)**	**Included articles (n)**	**Intervention component/s (n)**	**Actors involved (n)**	**Target group/s (n)**	**Setting where intervention was provided (n)**	**Sample size (n)**
33	165*	Number of intervention components: • One (132) • Several (33) Types of intervention components: • Screening (100) • Counselling/Education (68) • Referral/Invitation (17) • Social activity (9) • Skills training (4) • Practical support (3) • Motivator (2) • Vaccination (1) • Physical activity (1)	Number of actors: • One (82) • Multiple (44) • Not reported (39) Types of actors: • Nurse (35) • GP (33) • CHW (22) • Health worker (21) • Project staff (12) • Specialist MD (9) • Midwife (6) • Other professions (6) • Counsellor (3) • Pharmacy staff (3) • Supporting position (2) • Dental staff (1) • Other (19)	Number of target groups: • One (150) • Multiple (15) Types of target groups: • Children > 5 (44) • Pregnant/Postpartum (37) • Older adults (33) • Caregivers (26) • General Population (13) • Adults (13) • Risk group (12) • Women (8) • Minority group (7) • Migrants (7) • Children ≤ 5 (2) • Men (2)	Number of settings: • One (133) • Multiple (19) • Not reported (13) Types of settings: • Primary Care Clinic (117) • Home (28) • Perinatal/Child Healthcare Clinic (7) • Community (7) • School (3) • Pharmacy (3) • Other (4)	• < 100 (36) • 100–999 (67) • 1000–10,000 (31) • > 10,000 (1) • Not reported (30)

*CHW* Community Health Worker, *GP* General Practitioner, *MD* Medical Doctor, *N* Number of reviews, *n* Number of articles; *5–15% duplicates

**Table 10:** Target area Communicable diseases

Reviews (N)	Included articles (n)	Intervention component/s (n)	Actors involved (n)	Target group/s (n)	Setting where intervention was provided (n)	Sample size (n)
16	99*	Number of interventions: • One (58) • Multiple (41) Types of interventions: • Counselling/Education (48) • Screening (41) • Reminder (19) • Referral/Invitation (16) • Incentive (9) • Pharmacology/Nutrition (6) • Vaccination (6) • Mobilisation (5) • Media (4)	Number of actors: • One (53) • Multiple (11) • Not reported (35) Types of actors: • CHW (31) • GP (17) • Nurse (12) • Other professions (5) • Health worker (2) • Project staff (1) • Counsellor (1) • Pharmacy staff (1) • Other (6)	Number of target groups: • One (92) • Multiple (7) Types of target groups: • Older adults (36) • General population (23) • Pregnant/Postpartum (15) • Children ≤ 5 (10) • Adults (8) • Migrants (5) • Minority (4) • Men (1) • Women (1) • Children > 5 (1) • Risk group (1)	Number of settings: • One (74) • Multiple (22) • Not reported (3) Types of settings: • Primary Care Clinic (55) • Home (28) • Community (22) • Perinatal/Child Healthcare Clinic (11) • Sexual Healthcare Clinic (1) • Pharmacy (1)	• < 100 (4) • 100–999 (29) • 1000–10,000 (24) • > 10,000 (21) • Not reported (21)

*CHW* Community Health Worker, *GP* General Practitioner, *N* Number of reviews, *n* Number of articles, *5–15% duplicates

**Table 11:** Target area Injuries, accidents and violence

**Reviews (N)**	**Included articles (n)**	**Intervention component/s (n)**	**Actors involved (n)**	**Target group/s (n)**	**Setting where intervention was provided (n)**	**Sample size (n)**
17	98*	Number of intervention components: • One (53) • Multiple (45) Types of intervention components: • Counselling/Education (67) • Screening (44) • Referral/Invitation (25) • Physical Activity (9) • Practical Support (9) • Incentive (9) • Pharmacology/Nutrition (3) • Motivator (1) • Social Activity (1)	Number of actors: • One (49) • Multiple (14) • Not reported (35) Types of actors: • Nurse (34) • GP (7) • Counsellor (6) • Supporting Position (6) • Midwife (4) • CHW (3) • Other Professions (3) • Specialist MD (1) • Health Workers (1) • Project Staff (1) • Other (11)	Number of target groups: • One (94) • Multiple (4) Types of target groups: • Pregnant/Postpartum (38) • Women (22) • Older Adults (18) • Caregivers (16) • Children > 5 (7) • Children** ≤ **5 (4) • Minority Group (4) • Risk Group (4) • Adults (2) • General Population (1) • Men (1)	Number of settings: • One (75) • Multiple (17) • Not reported (6) Types of settings: • Primary Care Clinic (40) • Perinatal/Child Healthcare Clinic (31) • Home (30) • Sexual Healthcare Clinic (4) • School (2) • Community (1) • Other (2)	• < 100 (5) • 100–999 (55) • 1000–10,000 (10) • Not reported (28)

*CHW* Community Health Worker, *GP* General Practitioner, *MD* Medical Doctor, *N* Number of reviews, *n* Number of articles, *5–15% duplicates

**Table 12:** Target area Oral health

Reviews (N)	Included articles (n)	Intervention component/s (n)	Actors involved (n)	Target group/s (n)	Setting where intervention was provided (n)	Sample size (n)
7	62*	Number of intervention components: • One (20) • Several (42) Types of intervention components: • Counselling/Education (59) • Incentive (20) • Referral/Invitation (15) • Screening (11) • Pharmacology/Nutrition (7) • Reminder (5) • Practical support (3) • Media (2) • Vaccination (1)	Number of actors: • One (40) • Multiple (18) • Not reported (4) Types of actors: • CHW (28) • Nurse (25) • GP (6) • Dental staff (6) • Health worker (5) • Other professions (4) • Counsellor (2) • Supporting position (1) • Specialist MD (1) • Midwife (1) • Other (2)	Number of target groups: • One (28) • Multiple (34) Types of target groups: • Caregivers (42) • Children ≤ 5 (39) • Women (14) • Children > 5 (12) • Minority group (12) • Pregnant/Postpartum (5) • General Population (4) • Risk group (2) • Older Adults (1)	Number of settings: • One (33) • Multiple (12) • Not reported (17) Types of settings: • Home (18) • Primary Care Clinic (16) • Perinatal/Child Healthcare Clinic (10) • Dental Clinic (8) • Community (6) • School (1) • Other (1)	• < 100 (5) • 100–999 (25) • 1000–10,000 (9) • > 10,000 (1) • Not reported (22)

*CHW* Community Health Worker, *GP* General Practitioner, *MD* Medical Doctor, *N* Number of reviews, *n* Number of articles, *5–15% duplicates

**Table 13:** Target area Drugs and other substances

Reviews (N)	Included articles (n)	Intervention component/s (n)	Actors involved (n)	Target group/s (n)	Setting where intervention was provided (n)	Sample size (n)
5	25*	Number of interventions: • One (19) • Multiple (6) Types of interventions: • Counselling/Education (25) • Screening (5) • Pharmacology/Nutrition (1)	Number of actors: • One (13) • Not reported (12) Types of actors: • GP (4) • Counsellor (4) • Nurse (2) • Project staff (2) • Supporting Position (1)	Number of target groups: • One (20) • Multiple (5) Types of target groups: • Children > 5 (14) • Risk group (14) • Adults (9) • Elderly (7)	Number of settings: • One (24) • Multiple (2) Types of settings: • Primary Care Clinic (23) • School (1) • Other (3)	• < 100 (2) • 100–999 (18) • 1000–10,000 (4) • Not reported (1)

*CHW* Community Health Worker, *GP* General Practitioner, *N* Number of reviews, *n* Number of articles, *20–25% duplicates

**Table 14:** Target area Diseases/conditions mostly affecting elderly

Reviews (N)	Included articles (n)	Intervention component/s (n)	Actors involved (n)	Target group/s (n)	Setting where intervention was provided (n)	Sample size (n)
8	24*	Number of intervention components: • One (11) • Multiple (13) Types of intervention components: • Screening (17) • Counselling/Education (13) • Referral/Invitation (9) • Physical Activity (2) • Practical Support (1)	Number of actors: • One (19) • Multiple (2) • Not reported (3) Types of actors: • Nurse (11) • GP (6) • Pharmacy staff (4) • Specialist MD (1) • Project staff (1)	Number of target groups: • One (21) • Multiple (3) Types of target groups: • Older Adults (21) • Adults (4) • Minority Group (1)	Number of settings: • One (23) • Not Reported (1) Types of settings: • Primary Care Clinic (12) • Home (7) • Pharmacy (3) • Community (1)	• < 100 (2) • 100–999 (11) • 1000–10,000 (8) • Not reported (3)

*GP* General Practitioner, *MD* Medical Doctor, *N* Number of reviews, *n* Number of articles, *5–15% duplicates

**Table 15:** Target area Other

Reviews (N)	Included articles (n)	Intervention component/s (n)	Actors involved (n)	Target group/s (n)	Setting where intervention was provided (n)	Sample size (n)
7	58*	Number of intervention components: • One (35) • Multiple (23) Types of intervention components: • Counselling/Education (53) • Screening (12) • Referral/Invitation (10) • Reminder (3) • Practical support (3) • Pharmacology/Nutrition (3) • Incentive (2) • Social Activity (1)	Number of actors: • One (38) • Multiple (11) • Not reported (9) Types of actors: • Nurse (20) • GP (13) • CHW (11) • Other professions (6) • Specialist MD (5) • Counsellor (3) • Health worker (2) • Midwife (1) • Project staff (1) • Other (3)	Number of target groups: • One (57) • Multiple (1) Types of target groups: • Caregivers (25) • Risk group (12) • Older Adults (10) • Pregnant/Postpartum (10) • General Population (8) • Women (8) • Adults (5) • Minority group (2) • Migrants (2)	Number of settings: • One (47) • Multiple (7) • Not reported (4) Types of settings: • Perinatal/Child Healthcare Clinic (21) • Primary Care Clinic (20) • Home (15) • Community (1) • Other (4)	• < 100 (8) • 100–999 (29) • 1000–10,000 (9) • > 10,000 (1) • Not reported (11)

*CHW* Community Health Worker, *GP* General Practitioner, *MD* Medical Doctor, *N* Number of reviews, *n* Number of articles, *5–15% duplicates

#### Cardiovascular disease/Diabetes

Within the target area Cardiovascular disease/Diabetes, a total of 1,044 articles were included. Each intervention contained between one and four intervention components, with single-component interventions being most common. *Counselling/Education* and *Screening* were the most frequent components. Within the *Counselling/Education* category*,* interventions consisted of brief advice or motivational interviewing for smoking cessation, healthy diet, exercise, or alcohol consumption. *Screening* interventions primarily involved assessment for hazardous alcohol consumption or general health evaluations for cardiovascular disease risk. When information about the actor providing the intervention was available, most interventions were delivered by a single type of provider. *GP* was the most common provider, followed by *Nurse* and *CHW*. Most interventions targeted one population group. The most frequently targeted groups were *Adults* and individuals in *Risk groups* because of hazardous alcohol consumption or tobacco use. Regarding intervention setting, most interventions were delivered in one setting, with the most common being the *Primary Care Clinic*, followed by the participant’s *Home*. Most articles had a sample size between 100 and 999 participants. The top five countries where interventions were provided: USA (326); UK (185); Australia (47); Spain (43); and Sweden (30).

#### Perinatal and child health

Within the Perinatal and child health area, 378 articles were included. Each intervention had between one and five components, with two thirds of the articles being single-component interventions. The most used component was *Counselling/Education* followed by *Screening,* and *Referral/Invitation*. The type of *Counselling/Education* delivered varied, but interventions mostly entailed providing information and/or discussing various topics related to perinatal and child health with individuals, families, or groups. *Screening* interventions involved, for example, assessing health status and identifying potential risk factors in pregnant women, growth assessments among children, or conducting social needs assessments for families. Most intervention components were delivered by one actor, who was, most commonly, a CHW. The intervention components were usually delivered to a single population group. *Pregnant/Postpartum* women were mostly targeted followed by *Caregivers*, *Children* ≤ *5* and *Women*. In 41 articles, interventions focused on a specific *Risk group*, predominantly individuals with low socio-economic status. The setting of most interventions was the *Home* followed by *Primary Care Clinic* and *Perinatal/Child Healthcare Clinic*. The top five countries where interventions were provided: USA (82); India (31); Pakistan (27); Bangladesh (26); and Australia (24).

#### Sexual and reproductive health

Within the target area Sexual and reproductive health 239 articles were analysed. Each intervention provided between one and four intervention components, with single-component interventions, such as *Counselling/Education* and *Screening,* being the most frequent. The category *Counselling/Education* included interventions providing one-on-one counselling or educational material about sexually transmitted diseases, and contraception. *Screening* was conducted at home or in a clinic for sexually transmitted diseases. Among articles providing information on intervention actors, a single type of actor was with *CHW* and *Counsellor* being mentioned most often, followed by *Nurse* and *GP*. In most articles, a single population group was targeted, most frequently *Children* > *5* and *Adults*. All but 13 articles specified the intervention setting, the most common being a *Sexual Healthcare Clinic* followed by a *Primary Care Clinic*. The top five countries where interventions were provided: USA (127); Australia (18); South Africa (10); UK (7); and Malawi (6).

#### Cancer

The target area *Cancer* included 235 articles. Both single- and multiple- component interventions were described, with between one and five components delivered. *Counselling/Education* and *Reminders* were provided most often, but *Screening*, *Referral/Invite* and *Practical support* were also common. *Counselling/Education* involved e.g., providing information about cancer, screening procedures, and how to self-examine. *Reminders* were sent out to individuals who had missed screening, and invitations were sent out to encourage screening. *Screening* kits were also provided in some interventions, and support was given to enable people to attend screening. Most interventions were provided by a single actor, the most frequent being a *CHW* followed by a *GP*. Interventions were most often delivered to a single target group, however addressing the health concerns of multiple groups in one intervention was also common. The target groups varied; however, *Adults*, *Women,* and *Minority groups were the largest*. Specific risk groups consisted of individuals with low socio-economic position, individuals using alcohol/tobacco, and other groups with specific risk for cancer. Most articles described interventions that had been carried out in a single setting. The most common was the *Primary Care Clinic*, followed by *Home* and the *Community*. Top five countries where interventions were provided: USA (166); UK (20); Italy (8); Australia (12); and Canada (3).

#### Mental health

Target area 5 (Mental Health) comprised information from 165 articles. Between one and three components were provided in the interventions, the majority being single-component interventions. *Screening* was the most common component, followed by *Counselling/Education,* and *Referral/Invitation*. *Screening* for depression, anxiety, or loneliness was recurrent, followed by *Counselling* in the form of Cognitive Behavioural Therapy or individual/group sessions to reduce stress and depression, or boost active lifestyles. Examples from the category *Referral* included linkage to community resources and activities, and referral for treatment. Most interventions were delivered by a single actor the most common being a *Nurse,* followed by a *GP,* and a *CHW* or a *Health worker*. The majority of interventions were aimed at one target group, with the most common being *Children* > *5* and their *Caregivers*, *Pregnant/Postpartum* women, and *Older adults*. The category *Risk groups* encompassed individuals with mental health problems, low socio-economic position, or alcohol or drug users. Most articles described interventions that had been carried out in a single setting, predominantly the *Primary Care Clinic*. Other settings such the participants’ *Home*, the *Perinatal/Child healthcare clinic*, the *Community* and the *School* were also mentioned. Top five countries where interventions were provided: USA (44); UK (16); Spain (11); Japan (7); and Norway/South Africa/Netherlands (5).

#### Communicable diseases

Within target area 6 (Communicable diseases), 99 articles were included. Interventions provided between one and four intervention components. Single-component interventions were most common, with *Counselling/Education* and *Screening* being frequently used. Examples within the category *Counselling/Education* are one-on-one counselling sessions or education or informational brochures about vaccination. Examples within the category *Screening* are doorstep detection of tuberculosis or Hepatitis testing in clinics. Among articles presenting information about the intervention provider, single-actor interventions were the most frequent. Interventions were generally provided by a *CHW* or *GP*. Interventions were usually delivered to one population group, with the most common group being *Older adults,* followed by *General population*. All articles but three described the intervention setting, in most cases this being a *Primary Care Clinic*, followed by the participants’ *Home,* or in the *Community*. Top five countries where interventions were provided: USA (15); UK (14); Canada (10); Ethiopia (6); and Gambia (6).

#### Injuries, accidents and violence

Within target area 7 (Injuries, accidents, and violence), 98 articles were included. In these, one to five intervention components were provided. One and multiple intervention components were used to a similar extent. *Counselling/Education* was the most used intervention component. This component varied in format; it could, for example, be an educational video shown for child injury prevention, repeated counselling sessions in a program provided over time or offered access to counselling concerning intimate partner violence. Another common intervention component was *Screening*, which, for example, could be home safety assessment or intimate partner violence screening. Among the articles having information about different types of actors (*n* = 63) it was most common with one actor (*n* = 49). Many different actors were used in this target area. However, *Nurse* was by far the most common actor. Mostly often one target group was approached, with *Pregnant/Postpartum* women being the largest group. Both one and multiple settings were identified, where *Primary Care Clinic*, *Perinatal/Child Healthcare Clinic* and *Home* where the most common settings. Top five countries where interventions were provided: USA (47); Canada (5); Australia (4); Netherlands (3); and New Zealand (3).

#### Oral health

Within target area 8 (Oral Health), 62 articles were included. Interventions provided included between one to four components. Most interventions described contained several components. *Counselling/Education* was the most common component, followed by *Incentives*. *Counselling/Education* was, for example, oral health education on preventive dental care, and incentives were often gifts such as toothbrushes and fluoride toothpaste. There were also several examples of *Screening* for dental caries and encouraging people to seek dental care. In most interventions, one actor was involved in the intervention, the most common being *CHW*, followed by *Nurse, GP* and *Dental staff*. It was approximately as common to have one as multiple target groups. There were examples on many different target groups, the major being *Caregivers* and *Children at the age of five or under*. The interventions had been carried out in both single and multiple settings. Both clinical settings such as *Primary Care Clinic, Perinatal/Child Healthcare Clinic* and *Dental Clinics* were described, as well as non-clinical settings such as *Home* and the *Community*. Top five countries where interventions were provided: USA (17); Australia (7); Canada (6); UK (5); and India (5).

#### Drugs and other substances

Within target area 9 (Drugs and other substances), including 25 articles, between one and two intervention components were provided. The most common was to give a single component intervention and the most frequently used type was *Counselling/Education.* Examples within this were motivational interviewing, brief interventions, and educational programmes about the risks of substance abuse. About half of the articles reported on the actor, mostly with one actor providing the intervention alone and most often being a GP or Counsellor. In most articles, one population group was targeted, most common was *Children* > *5* and *Risk groups* because of alcohol or drug use. The intervention component was primarily provided at a *Primary Care Clinic.* Top five countries where interventions were provided: USA (17); Multiple countries (3); UK (2); and Canada/France/Switzerland (1).

#### Conditions mostly affecting elderly

Within target area 10 (Conditions mostly affecting elderly), including 24 articles, one to three intervention components were provided. It was almost as common to give multiple components as a single component. The most used component was *Screening*, e.g., cognitive assessments, visual screening and various risk assessments. It was most common to use one actor providing the intervention component(s) within this target area, e.g. *Nurse*, *GP*, or *Pharmacist*. In most articles one population group was targeted – *Older adults* the commonest, but also *Adults* on some occasions, and a *Minority Group* in one article (lacking age information). The intervention component was primarily provided at a *Primary Care Clinic* or at *Home*. Top five countries where interventions were provided: UK (11); USA (5); Netherlands (3); Canada (2); and Spain (2).

#### Other

Target area 11 (Other) included 58 articles on prevention of exposure to passive smoking (32), care needs assessments (18), and organ donation promotion (8). Both single and multiple component interventions were described with between one and four components delivered. *Counselling/Education* was the most common component, but *Screening* and *Referral/Invitation* were also described in several articles. *Counselling/Education* involved brief advice and written information regarding on e.g., how to reduce child exposure to tobacco smoke or how to sign up for organ donation. *Screening* included measures of smoke exposure or assessments of care needs. Referral was for example being referred to quit line when smoking. In most interventions, one actor was involved in the intervention. The most common actor groups were *Nurse, GP* and *CHW*. Almost all interventions were aimed at one target group only, the largest groups being *Caregivers*, *Risk Groups* (i.e. smokers), *Older adults*, and *Pregnant/Postpartum* women. Most articles described interventions carried out in a single setting. The most common was *Perinatal/child healthcare*, *Primary Care Clinic*, and *Home*. Top five countries where interventions were provided: USA (25); UK (8); Australia (4); Canada (3); and Italy (3).

## Discussion

This scoping review of reviews mapped population-oriented health promotion and disease prevention interventions within primary healthcare. The interventions were provided in all six regions of the World Health Organization, with the vast majority in high-income countries. The interventions targeted varying health issues, e.g., diabetes, cancer, mental health, and violence. The most common areas addressed by interventions were cardiovascular disease and diabetes, where many focused on prevention and promotion through targeting lifestyle habits. The *GP* was the category of actors who provided the largest number of interventions, and the *Adult population* was the largest target group. The interventions were most provided in the *Primary Care Clinic*. Thus, it seems that many health promotion and disease prevention interventions have been delivered within clinical settings, often on an individual basis. There were, however, differences regarding intervention components, actors, settings, and target groups across the different target areas. Some of these differences are discussed below.

### Interventions

The intervention component *Counselling/Education* was most used. This is a broad category, as it includes everything from shorter brochures to intensive one-on-one counselling applicable to all target areas. Counselling and education are also strategies that are deeply embedded in the idea of health promotion information and disease prevention, which, by definition, largely focuses on educating people on health risks and empowering them to take control of their own health [23, 339]. *Screening* was the second most common component, containing activities such as testing for sexually transmitted diseases, cancer screening, and general health assessments. *Referral/Invitation* and *Reminder* were also frequently used, often as part of multi-component interventions and applicable across all target areas. Counselling, education, and screening are common but not necessarily the most effective components. Their prevalence may stem from their versatility in various settings and the inclusion of low-intensity interventions like brief advice and questionnaires, which require minimal time and resources. This review highlights opportunities to explore new and potentially more effective strategies in future intervention studies.

### Target group

The identified target groups varied based on the intervention goals*. Pregnant/Postpartum* women were the third largest target group for all types of interventions and represented the most prominent target group within *Perinatal and child health* and within *Injuries, accidents and violence*. The prominence within the latter target area might be explained by screenings for intimate partner violence in check-ups during pregnancy [340]. Globally, during the last two to three decades, increased attention has been given to both newborn children and pregnant/postpartum women, e.g. by implementing simple and evidence-based interventions, such as family planning, maternal immunisation, maternal interventions to improve psychosocial health and substance abuse, and exclusive breastfeeding of the newborn child [341, 342], resulting in improved health and survival [341, 342]. The Sustainable Development Goals 3 and specifically targets 3.1 and 3.2 [343] have also enhanced awareness about maternal and newborn health, which our results probably mirror, as many interventions aimed at Pregnant/Postpartum women and newborn children are considered health-promoting and preventive. Interventions during the perinatal period might also include many of the target areas in this review, depending on the target group’s needs.

*Minority groups* stood out as one of the most common target group within *Cancer*. Minorities suffer disproportionately of cancer compared to the general population [344], making this demographic particularly important to target with preventive measures.

Many interventions aim to address the health concerns of particular *Risk Groups*. *Risk groups* were prevalent within areas concerning lifestyle habits, for instance, *Cardiovascular disease/Diabetes*, *Drugs and other substances* and the target group *Other* (which included prevention of child exposure to second-hand smoking). Risk groups are important targets for the prevention of unhealthy lifestyle habits, given that some poor lifestyle habits tend to cluster, such as smoking and alcohol use [345]. The least common target groups overall were *Migrants* and *Men*. This may be because many interventions targeted adults in general (including men and migrants), but it could also be an indication that these groups would benefit from being explicitly targeted in future health promoting and disease preventive interventions. For instance, the clustering of unhealthy lifestyle habits was associated with male gender in a previous review [345], and migrants have been found to have increased risk of both communicable and non-communicable diseases, as well as mental ill-health [346]. There are a number of barriers to seeking medical and psychological help among men [347] and to men’s participation in health research [348]. There is a lack of evidence on how to improve men’s involvement in health promotion efforts [349], although increasing health literacy has been suggested as one possible key [350]. This lack of male engagement could be considered not only a missed opportunity to improve men’s health but could also be a missed opportunity in utilizing men’s engagement in health promotion in their family settings. Further, there is some evidence that health interventions targeting migrants have been rather narrow in scope and might, therefore, not accurately reflect the morbidity and needs in immigrant groups [351]. Information on how target groups were invited or identified was scarce, only a fourth of the included articles had this information. For future studies, reporting this information could be helpful to those planning to implement interventions within primary healthcare.

### Actors

In this scoping review, we identified several types of actors providing the interventions (see Table 3). The most common types were actors who belong to, or are closely connected with, the *Primary care clinic*, such as *GP, CHW* and *Nurse*. But other actors within the primary healthcare setting, for example *Pharmacy staff* and *Dental clinic staff*, were also common.

Within the target areas *Drugs and other substances* and *Injuries, Accident and Violence*, the *Counsellor* was a common actor. A *Counsellor* is a trained professional that provides general and specialized support and therapeutical interventions to people experiencing a wide range of psychological, emotional, and behavioural problems [352]. Both *GPs* and *Nurses* commonly conduct counselling in their daily work. However, performing counselling effectively might require additional specialised efforts and skills [353]. Hence, a trained *Counsellor* might be better suited than a *GP* or a *Nurse* to handle certain problems [354]. Previous research shows positive results involving mental health counsellors in primary healthcare, making care more accessible to individuals [355]. In this review, interventions delivered by counsellors were mainly common in high-income countries.

In six of the 11 target areas (*Cardiovascular disease/Diabetes; Mental health; Injuries, accidents and violence; Drugs and other substances; Conditions mainly affecting the elderly;* and *Others*) the *Nurse* or *GP* was the most common actor, while in the remaining five target areas (*Perinatal and Child Health; Sexual and Reproductive Health; Cancer; Infectious Diseases;* and *Oral Health*) the *CHW* was the most common the actor. A *CHW* position in this review includes many different types of actors. However, classifying these actors as *CHWs* suggests that they are close to the population and often perform activities in the community or at people’s homes [356]. In this scoping review, the *CHW* role was most common in low- and middle-income countries. In one of the included reviews [128] it was concluded that CHWs are effective in prevention of maternal and child ill-health in low- and middle-income countries. Our review also contained some high-income countries using CHWs, such as Canada and the USA. In the USA, CHWs have been active for more than 70 years, and they have been highlighted as key in linking marginalised communities with health care and public health services [357].

Many high income countries, such as Sweden, have focused on transforming their healthcare services to be more person-centred [358]. This involves a more preventive approach on the part of healthcare staff [359]. However, the lack of resources has been a barrier to implementing health promotion and disease prevention interventions up to this point [360]. CHWs could help in bridging the gap between the healthcare system and the population by improving outreach in underserved and vulnerable communities.

### Setting

Regarding the distribution of countries where the interventions were carried out, USA was the most common country and represented in all target areas, followed by the UK and Australia. This reflects the high proportion of English-speaking public health research that is produced in these countries [361]. Within most target areas, the top five most common countries were all high-income. However, two exceptions were *Perinatal and child health* and *Communicable diseases*, where two or more of the top five countries were low-income and lower middle-income [362]. As non-communicable diseases are becoming a growing cause of morbidity and mortality in low-income and lower middle-income countries, our results highlight the need for more intervention studies within this field also outside of high-income countries [363]. The *Primary care clinic* was the most common setting overall in the data set, followed by the participant’s *Home* and *Perinatal/Child healthcare clinic*. As we looked for population-based interventions within primary healthcare and included population-based interventions in clinics, it is not surprising that *Primary care clinic* was the most common setting.

The frequent use of *Perinatal/Child healthcare clinic* as a setting, not only within *Perinatal and child health*, shows that it is a common option for a wide variety of preventive interventions targeting pregnant women and newly delivered children [364]. Interventions outside the clinic were primarily performed in participants’ *Homes* and less commonly in the *Community*. Our data could be affected by the fact that most included studies were randomised controlled trials, which are easier to perform in a clinic or a home, rather than in the community [365]. Regardless, our results highlight a need for more intervention studies in the community, as it is a crucial arena to reach parts of the population that do not come to the primary care clinic regularly and often are in the greatest need of preventive health measures. This is important to consider in the quest to achieve health equity, where access to health is a fundamental factor [366].

### Strengths and limitations

This work was a review of reviews, but focusing on articles presented in the reviews Results sections that met our inclusion criteria. The rationale for selecting articles from the reviews for the analysis, was that there were very few reviews that, in their entirety, contributed to the result of the current review. Hence, it was not possible to draw conclusions from the overall result of a review. Out of 8,361 articles (all articles in the result sections of the 310 reviews), we only selected 2,426 articles (those articles in result sections of the 310 reviews meeting our inclusion criteria) for analysis. It’s possible that this method implied that not all available original studies were identified as the descriptions of individual articles in the reviews were short, i.e. articles might have been excluded when the description was judged not meeting the inclusion criteria. Further, only reviews and articles written in English were included, which may be considered a limitation of this review. In addition, this method can imply a risk for duplicates, i.e., that several reviewers have captured the same articles, which we then present summaries of. We have detected between 5–15% duplicates in nine target areas and 20–25% duplicates in the remaining two areas, which need to be considered when interpreting the results. The strength of this approach, however, is that all included articles are relevant for the aim of this study and focus on promotive and preventive interventions in primary healthcare. An additional strength of the current method was our rigorous approach used in the review process. At least two independent reviewers were involved in all screening steps, supported by an author group that has been engaged in discussing and addressing problems on a weekly basis throughout the entire process of screening, data extraction, and analysis. While the process was time-intensive due to the comprehensive material, this rigorous approach ensures the reliability of the results.

## Conclusion

This scoping review of reviews offers an opportunity for a broad mapping of the interventions performed within primary healthcare, rather than a specific type of intervention or target area. Our results indicate that most health promotion and disease prevention interventions are still being delivered within clinical settings, often on individual basis. However, there were differences between the identified target areas regarding the most common intervention components, actors, target groups, and settings. The results may be used by various types of stakeholders, such as decision-makers and clinicians for further development of interventions and planning for implementation of interventions targeting a variety of groups, involving different types of actors, and provided in differing settings all within primary healthcare. It may also serve as an overview for researchers, of interventions provided within different health areas, and to what aspects of this phenomenon where more research is needed, for instance interventions targeting migrants, men and how CHW could be utilized in high-income countries.

## Supplementary Information

Supplementary Material 1.

Supplementary Material 2.

Supplementary Material 3.

Supplementary Material 4.

## Data Availability

All data generated or analysed during this study are included in this published article [and its additional files].

## Abbreviations

AFR: African Region
AMR: Regions of the Americas
CHW: Community Health Worker
EMR: Eastern Mediterranean
EUR: European Region
GP: General Practitioner
SEAR: South-East Asian Region
WPR: Western Pacific Region
