# Supplementary material for: Population-oriented health promotion and disease prevention interventions in primary healthcare: a scoping review of reviews
Source: BMC Prim Care. 2026 Apr 22;27:154. doi: 10.1186/s12875-026-03337-y (PMC13104254; doi:10.1186/s12875-026-03337-y)
Supplement: Supplementary file 3 — Supplementary Material 3. [file 12875_2026_3337_MOESM3_ESM.docx]

Additional file 2. Search strategies

Search strategy in Medline

Field labels

- exp/ = exploded MeSH term
- / = non exploded MeSH term
- .ti,ab,kf. = title, abstract and author keywords
- adjx = within x words, regardless of order
- * = truncation of word for alternate endings

| #1 Primary Health Care/ |
| --- |
| #2 General Practice/ |
| #3 exp Community Health Services/ |
| #4 Family Practice/ |
| #5 Primary Care Nursing/ |
| #6 exp Community Health Nursing/ |
| #7 Child Health Services/ |
| #8 Public Health Nursing/ |
| #9 Maternal-Child Health Centers/ |
| #10 Physicians, Primary Care/ |
| #11 (general practi* or family practi*).ti. |
| #12 (primary adj3 (care or health*)).ti. |
| #13 (communit* adj3 (based or care or health* or network* or nursing or outreach or participat* or service* or support)).ti. |
| #14 ((child* or maternal) adj3 health*).ti. |
| #15 or/1-14 |
| #16 "Public Health"/ or "Accidental Falls"/ or "Accident Prevention"/ or Accidents/ or "Accidents, Aviation"/ or "Accidents, Home"/ or "Accidents, Occupational"/ or "Accidents, Traffic"/ or "Air Microbiology"/ or "Air Pollution"/ or Biofouling/ or Biometry/ or "Body Burden"/ or Chemometrics/ or "Communicable Disease Control"/ or Decontamination/ or Demography/ or "Disease Eradication"/ or "Disease Hotspot"/ or "Disease Outbreaks"/ or "Disease Transmission, Infectious"/ or "Disease Vectors"/ or Drowning/ or Emergencies/ or "Endemic Diseases"/ or "Environmental Exposure"/ or "Environmental Medicine"/ or "Environmental Microbiology"/ or "Environmental Monitoring"/ or "Environmental Pollution"/ or "Environmental Restoration and Remediation"/ or Epidemics/ or "Epidemiologic Measurements"/ or "Epidemiologic Methods"/ or Epidemiology/ or "Food Contamination"/ or "Food Microbiology"/ or "Food Quality"/ or "Food Safety"/ or "Hand Hygiene"/ or "Health Transition"/ or Hygiene/ or "Infectious Disease Incubation Period"/ or "Infectious Disease Transmission, Vertical"/ or "Light Pollution"/ or "Mass Drug Administration"/ or "Mass Screening"/ or "Medical Countermeasures"/ or Noise/ or "Nutrition Assessment"/ or "Nutrition Surveys"/ or "Nutritive Value"/ or "Occupational Medicine"/ or "Petroleum Pollution"/ or Pharmacovigilance/ or "Population Surveillance"/ or "Preventive Medicine"/ or "Preventive Psychiatry"/ or "Primary Prevention"/ or "Public Health Practice"/ or "Quaternary Prevention"/ or "Risk Assessment"/ or "Secondary Prevention"/ or "Soil Microbiology"/ or "Space-Time Clustering"/ or "Tertiary Prevention"/ or "Traffic-Related Pollution"/ or "Waste Products"/ or "Water Microbiology"/ or "Water Pollution"/ |
| #17 (public health or accidental fall* or accident prevention or accident* or air microbiology or air pollution* or biofouling or biometry or body burden or chemometric* or communicable disease control or decontamination or demography or disease eradication or disease hotspot* or disease outbreak* or infectious disease transmission* or disease vector* or drowning or emergenc* or endemic disease* or environmental exposure or environmental medicine or environmental microbiology or environmental monitoring or environmental pollution or "environmental restoration and remediation" or epidemic* or epidemiologic measurement* or epidemiologic method* or epidemiology or food contamination or food microbiology or food quality or food safety or hand hygiene or health transition or hygiene or infectious disease incubation period or infectious disease transmission or light pollution or mass drug administration or mass screening* or medical countermeasure* or noise or nutrition assessment* or nutrition survey* or nutritive value* or occupational medicine or petroleum pollution or pharmacovigilance or population surveillance or preventive medicine or preventive psychiatry or primary prevention or public health practice* or quaternary prevention or risk assessment or secondary prevention or soil microbiology or spacetime clustering or tertiary prevention or trafficrelated pollution or waste product* or water microbiology or water pollution).ti,ab. |
| #18 Preventive Health Services/ or Diagnostic Services/ or Diagnostic Screening Programs/ or Mass Screening/ or Mobile Health Units/ or Early Intervention, Educational/ or Early Medical Intervention/ or Health Education/ or Consumer Health Information/ or Health Education, Dental/ or Health Fairs/ or Health Promotion/ or Smoking Prevention/ or Immunization Programs/ or Mass Vaccination/ or Primary Prevention/ or Immunization/ or Pre-Exposure Prophylaxis/ or Quaternary Prevention/ or Secondary Prevention/ or Tertiary Prevention/ or Vaccination Coverage/ |
| #19 (preventive health service* or diagnostic service* or diagnostic screening program* or mass screening* or mobile health unit* or early educational intervention or early medical intervention or health education or consumer health information or dental health education or health fair* or health promotion or smoking prevention or immunization program* or mass vaccination* or primary prevention or immunization or preexposure prophylaxis or quaternary prevention or secondary prevention or tertiary prevention or vaccination coverage).ti,ab. |
| #20 exp Health Promotion/ |
| #21 (intervent* or prevent* or promot* or program*).ti. |
| #22 (community health or health promot* or healthy people program* or preventive medicine or public health).ti,ab,kf. |
| #23 or/16-22 |
| #24 Meta-Analysis/ or "Systematic review"/ |
| #25 ((rapid or realist or systematic or scoping or umbrella) adj2 (review* or overview*)).ti,ab,kf. |
| #26 (meta-analy* or metaanaly* or metasynthe* or meta-synthe* or "realist synthes*" or "review* of reviews").ti,ab,kf. |
| #27 24 or 25 or 26 |
| #28 15 and 23 and 27 |

Search strategy in Web of Science

| Field labels   - TS/Topic = title, abstract, author keywords and Keywords Plus - NEAR/x = within x words, regardless of order - * = truncation of word for alternate endings   Note: the *Exact search*-function was used for all the searches |
| --- |

| 14 #5 AND #10 AND #13 |
| --- |
| 13 #11 OR #12 |
| 12 ((TI=(meta-analy* or metaanaly* or metasynthe* or meta-synthe* or "realist synthes*" or "review* of reviews")) OR AB=(meta-analy* or metaanaly* or metasynthe* or meta-synthe* or "realist synthes*" or "review* of reviews")) OR AK=(meta-analy* or metaanaly* or metasynthe* or meta-synthe* or "realist synthes*" or "review* of reviews") |
| 11 ((TI=(rapid or realist or systematic or scoping or umbrella NEAR review* or overview*)) OR AB=(rapid or realist or systematic or scoping or umbrella NEAR review* or overview*)) OR AK=(rapid or realist or systematic or scoping or umbrella NEAR review* or overview*) |
| 10 #6 OR #7 OR #8 OR #9 |
| 9 ((TI=("community health" OR "health promot*" OR "healthy people program*" OR "preventive medicine" OR "public health")) OR AB=("community health" OR "health promot*" OR "healthy people program*" OR "preventive medicine" OR "public health")) OR AK=("community health" OR "health promot*" OR "healthy people program*" OR "preventive medicine" OR "public health") |
| 8 TI=(intervent* or prevent* or promot* or program*) |
| 7 (TI=("preventive health service*" OR "diagnostic service*" OR "diagnostic screening program*" OR "mass screening*" OR "mobile health unit*" OR "early educational intervention" OR "early medical intervention" OR "health education" OR "consumer health information" OR "dental health education" OR "health fair*" OR "health promotion" OR "smoking prevention" OR "immunization program*" OR "mass vaccination*" OR "primary prevention" OR immunization OR "preexposure prophylaxis" OR "quaternary prevention" OR "secondary prevention" OR "tertiary prevention" OR "vaccination coverage")) OR AB=("preventive health service*" OR "diagnostic service*" OR "diagnostic screening program*" OR "mass screening*" OR "mobile health unit*" OR "early educational intervention" OR "early medical intervention" OR "health education" OR "consumer health information" OR "dental health education" OR "health fair*" OR "health promotion" OR "smoking prevention" OR "immunization program*" OR "mass vaccination*" OR "primary prevention" OR immunization OR "preexposure prophylaxis" OR "quaternary prevention" OR "secondary prevention" OR "tertiary prevention" OR "vaccination coverage") |
| 6 (TI=( ("public health" OR "accidental fall*" OR "accident prevention" OR accident* OR "air microbiology" OR "air pollution*" OR biofouling OR biometry OR "body burden" OR chemometric* OR "communicable disease control" OR decontamination OR demography OR "disease eradication" OR "disease hotspot*" OR "disease outbreak*" OR "infectious disease transmission*" OR "disease vector*" OR drowning OR emergenc* OR "endemic disease*" OR "environmental exposure" OR "environmental medicine" OR "environmental microbiology" OR "environmental monitoring" OR "environmental pollution" OR "environmental restoration and remediation" OR epidemic* OR "epidemiologic measurement*" OR "epidemiologic method*" OR epidemiology OR "food contamination" OR "food microbiology" OR "food quality" OR "food safety" OR "hand hygiene" OR "health transition" OR hygiene OR "infectious disease incubation period" OR "infectious disease transmission" OR "light pollution" OR "mass drug administration" OR "mass screening*" OR "medical countermeasure*" OR noise OR "nutrition assessment*" OR "nutrition survey*" OR "nutritive value*" OR "occupational medicine" OR "petroleum pollution" OR pharmacovigilance OR "population surveillance" OR "preventive medicine" OR "preventive psychiatry" OR "primary prevention" OR "public health practice*" OR "quaternary prevention" OR "risk assessment" OR "secondary prevention" OR "soil microbiology" OR "spacetime clustering" OR "tertiary prevention" OR "trafficrelated pollution" OR "waste product*" OR "water microbiology" OR "water pollution"))) OR AB=( ("public health" OR "accidental fall*" OR "accident prevention" OR accident* OR "air microbiology" OR "air pollution*" OR biofouling OR biometry OR "body burden" OR chemometric* OR "communicable disease control" OR decontamination OR demography OR "disease eradication" OR "disease hotspot*" OR "disease outbreak*" OR "infectious disease transmission*" OR "disease vector*" OR drowning OR emergenc* OR "endemic disease*" OR "environmental exposure" OR "environmental medicine" OR "environmental microbiology" OR "environmental monitoring" OR "environmental pollution" OR "environmental restoration and remediation" OR epidemic* OR "epidemiologic measurement*" OR "epidemiologic method*" OR epidemiology OR "food contamination" OR "food microbiology" OR "food quality" OR "food safety" OR "hand hygiene" OR "health transition" OR hygiene OR "infectious disease incubation period" OR "infectious disease transmission" OR "light pollution" OR "mass drug administration" OR "mass screening*" OR "medical countermeasure*" OR noise OR "nutrition assessment*" OR "nutrition survey*" OR "nutritive value*" OR "occupational medicine" OR "petroleum pollution" OR pharmacovigilance OR "population surveillance" OR "preventive medicine" OR "preventive psychiatry" OR "primary prevention" OR "public health practice*" OR "quaternary prevention" OR "risk assessment" OR "secondary prevention" OR "soil microbiology" OR "spacetime clustering" OR "tertiary prevention" OR "trafficrelated pollution" OR "waste product*" OR "water microbiology" OR "water pollution")) |
| 5 #4 OR #3 OR #2 OR #1 |
| 4 TI=((communit* NEAR/2 (based OR care OR health* OR network* OR nursing OR outreach OR participat* OR service* OR support))) |
| 3 TI=((child* or maternal) NEAR/2 health*) |
| 2 TI=(primary NEAR/2 (care or health*)) |
| 1 TI=("general practi*" or "family practi*") |

Search strategy in Psycinfo

Field labels

- DE = subject heading
- TI = title
- AB = abstract
- KW = author keywords
- Nx = within x words, regardless of order
- * = truncation of word for alternate endings

| S22 S9 AND S17 AND S21 |
| --- |
| S21 S18 OR S19 OR S20 |
| S20 TI ( meta-analy* or metaanaly* or metasynthe* or meta-synthe* or "realist synthes*" or "review* of reviews" ) OR AB ( meta-analy* or metaanaly* or metasynthe* or meta-synthe* or "realist synthes*" or "review* of reviews" ) OR KW ( meta-analy* or metaanaly* or metasynthe* or meta-synthe* or "realist synthes*" or "review* of reviews" ) |
| S19 TI ( (rapid or realist or systematic or scoping or umbrella) N2 (review* or overview*) ) OR AB ( (rapid or realist or systematic or scoping or umbrella) N2 (review* or overview*) ) OR KW ( (rapid or realist or systematic or scoping or umbrella) N2 (review* or overview*) ) |
| S18 DE "Literature Review" OR DE "Systematic Review" |
| S17 S10 OR S11 OR S12 OR S13 OR S14 OR S15 OR S16 |
| S16 TI ( ("community health" or "health promot*" or "healthy people program*" or "preventive medicine" or "public health") ) OR AB ( ("community health" or "health promot*" or "healthy people program*" or "preventive medicine" or "public health") ) OR KW ( ("community health" or "health promot*" or "healthy people program*" or "preventive medicine" or "public health") OR AB ("community health" or "health promot*" or "healthy people program*" or "preventive medicine" or "public health") ) OR AB ( ("community health" or "health promot*" or "healthy people program*" or "preventive medicine" or "public health") ) OR KW ( ("community health" or "health promot*" or "healthy people program*" or "preventive medicine" or "public health") OR KW ("community health" or "health promot*" or "healthy people program*" or "preventive medicine" or "public health") ) OR AB ( ("community health" or "health promot*" or "healthy people program*" or "preventive medicine" or "public health") ) OR KW ( ("community health" or "health promot*" or "healthy people program*" or "preventive medicine" or "public health")) |
| S15 TI (intervent* or prevent* or promot* or program*) |
| S14 DE "Health Promotion" |
| S13 (TI=(("preventive health service*" OR "diagnostic service*" OR "diagnostic screening program*" OR "mass screening*" OR "mobile health unit*" OR "early educational intervention" OR "early medical intervention" OR "health education" OR "consumer health information" OR "dental health education" OR "health fair*" OR "health promotion" OR "smoking prevention" OR "immunization program*" OR "mass vaccination*" OR "primary prevention" OR immunization OR "preexposure prophylaxis" OR "quaternary prevention" OR "secondary prevention" OR "tertiary prevention" OR "vaccination coverage" )OR AB=(("preventive health service*" OR "diagnostic service*" OR "diagnostic screening program*" OR "mass screening*" OR "mobile health unit*" OR "early educational intervention" OR "early medical intervention" OR "health education" OR "consumer health information" OR "dental health education" OR "health fair*" OR "health promotion" OR "smoking prevention" OR "immunization program*" OR "mass vaccination*" OR "primary prevention" OR immunization OR "preexposure prophylaxis" OR "quaternary prevention" OR "secondary prevention" OR "tertiary prevention" OR "vaccination coverage")) |
| S12 DE "Preventive Health Services" OR DE "Preventive Mental Health Services" OR DE "Prophylactic Drug Therapy" |
| S11 TI ( "public health" OR "accidental fall*" OR "accident prevention" OR accident* OR "air microbiology" OR "air pollution*" OR biofouling OR biometry OR "body burden" OR chemometric* OR "communicable disease control" OR decontamination OR demography OR "disease eradication" OR "disease hotspot*" OR "disease outbreak*" OR "infectious disease transmission*" OR "disease vector*" OR drowning OR emergenc* OR "endemic disease*" OR "environmental exposure" OR "environmental medicine" OR "environmental microbiology" OR "environmental monitoring" OR "environmental pollution" OR "environmental restoration and remediation" OR epidemic* OR "epidemiologic measurement*" OR "epidemiologic method*" OR epidemiology OR "food contamination" OR "food microbiology" OR "food quality" OR "food safety" OR "hand hygiene" OR "health transition" OR hygiene OR "infectious disease incubation period" OR "infectious disease transmission" OR "light pollution" OR "mass drug administration" OR "mass screening*" OR "medical countermeasure*" OR noise OR "nutrition assessment*" OR "nutrition survey*" OR "nutritive value*" OR "occupational medicine" OR "petroleum pollution" OR pharmacovigilance OR "population surveillance" OR "preventive medicine" OR "preventive psychiatry" OR "primary prevention" OR "public health practice*" OR "quaternary prevention" OR "risk assessment" OR "secondary prevention" OR "soil microbiology" OR "spacetime clustering" OR "tertiary prevention" OR "trafficrelated pollution" OR "waste product*" OR "water microbiology" OR "water pollution") OR AB ( "public health" OR "accidental fall*" OR "accident prevention" OR accident* OR "air microbiology" OR "air pollution*" OR biofouling OR biometry OR "body burden" OR chemometric* OR "communicable disease control" OR decontamination OR demography OR "disease eradication" OR "disease hotspot*" OR "disease outbreak*" OR "infectious disease transmission*" OR "disease vector*" OR drowning OR emergenc* OR "endemic disease*" OR "environmental exposure" OR "environmental medicine" OR "environmental microbiology" OR "environmental monitoring" OR "environmental pollution" OR "environmental restoration and remediation" OR epidemic* OR "epidemiologic measurement*" OR "epidemiologic method*" OR epidemiology OR "food contamination" OR "food microbiology" OR "food quality" OR "food safety" OR "hand hygiene" OR "health transition" OR hygiene OR "infectious disease incubation period" OR "infectious disease transmission" OR "light pollution" OR "mass drug administration" OR "mass screening*" OR "medical countermeasure*" OR noise OR "nutrition assessment*" OR "nutrition survey*" OR "nutritive value*" OR "occupational medicine" OR "petroleum pollution" OR pharmacovigilance OR "population surveillance" OR "preventive medicine" OR "preventive psychiatry" OR "primary prevention" OR "public health practice*" OR "quaternary prevention" OR "risk assessment" OR "secondary prevention" OR "soil microbiology" OR "spacetime clustering" OR "tertiary prevention" OR "trafficrelated pollution" OR "waste product*" OR "water microbiology" OR "water pollution") |
| S10 DE "Public Health" OR DE "Community Health" OR DE "Community Mitigation" OR DE "Public Health Attitudes" OR DE "Public Health Campaigns" OR DE "Public Health Research" OR DE "Public Mental Health" |
| S9 S1 OR S2 OR S3 OR S4 OR S5 OR S6 OR S7 OR S8 |
| S8 TI ((child* or maternal) N3 health*) |
| S7 TI (communit* N3 (based or care or health* or network* or nursing or outreach or participat* or service* or support)) |
| S6 TI (primary N3 (care OR health* )) |
| S5 TI ("general practi*" OR "family practi*" ) |
| S4 MM "Public Health Service Nurses" |
| S3 MM "Community Health" OR MM "Community Mental Health" |
| S2 MM "General Practitioners" |
| S1 MM "Primary Health Care" |

Search strategy in Cinahl

Field labels

- MH+ = exploded Cinahl Heading
- MH = non exploded Cinahl Heading
- TI = title
- AB = abstract
- Nx = within x words, regardless of order

* = truncation of word for alternate endings

| S27 | S13 AND S21 AND S26 |
| --- | --- |
| S26 | S22 OR S23 OR S24 OR S25 |
| S25 | TI ( ("meta-analy*" or metaanaly* or metasynthe* or "meta-synthe*" or "realist synthes*" or "review* of reviews") ) OR AB ( ("meta-analy*" or metaanaly* or metasynthe* or "meta-synthe*" or "realist synthes*" or "review* of reviews") ) |
| S24 | TI ((rapid OR realist OR systematic OR scoping OR umbrella ) N2 (review* OR overview* )) OR AB ((rapid OR realist OR systematic OR scoping OR umbrella ) N2 (review* OR overview* )) |
| S23 | (MH "Meta Analysis") |
| S22 | (MH "Scoping Review") OR (MH "Systematic Review") |
| S21 | S14 OR S15 OR S16 OR S17 OR S18 OR S19 OR S20 |
| S20 | TI ( "community health" OR "health promot*" OR "healthy people program*" OR "preventive medicine" OR "public health" ) OR AB ( "community health" OR "health promot*" OR "healthy people program*" OR "preventive medicine" OR "public health" ) |
| S19 | TI (intervent* or prevent* or promot* or program*) |
| S18 | TI ( "public health" OR "accidental fall*" OR "accident prevention" OR accident* OR "air microbiology" OR "air pollution*" OR biofouling OR biometry OR "body burden" OR chemometric* OR "communicable disease control" OR decontamination OR demography OR "disease eradication" OR "disease hotspot*" OR "disease outbreak*" OR "infectious disease transmission*" OR "disease vector*" OR drowning OR emergenc* OR "endemic disease*" OR "environmental exposure" OR "environmental medicine" OR "environmental microbiology" OR "environmental monitoring" OR "environmental pollution" OR "environmental restoration and remediation" OR epidemic* OR "epidemiologic measurement*" OR "epidemiologic method*" OR epidemiology OR "food contamination" OR "food microbiology" OR "food quality" OR "food safety" OR "hand hygiene" OR "health transition" OR hygiene OR "infectious disease incubation period" OR "infectious disease transmission" OR "light pollution" OR "mass drug administration" OR "mass screening*" OR "medical countermeasure*" OR noise OR "nutrition assessment*" OR "nutrition survey*" OR "nutritive value*" OR "occupational medicine" OR "petroleum pollution" OR pharmacovigilance OR "population surveillance" OR "preventive medicine" OR "preventive psychiatry" OR "primary prevention" OR "public health practice*" OR "quaternary prevention" OR "risk assessment" OR "secondary prevention" OR "soil microbiology" OR "spacetime clustering" OR "tertiary prevention" OR "trafficrelated pollution" OR "waste product*" OR "water microbiology" OR "water pollution" ) OR AB ( "public health" OR "accidental fall*" OR "accident prevention" OR accident* OR "air microbiology" OR "air pollution*" OR biofouling OR biometry OR "body burden" OR chemometric* OR "communicable disease control" OR decontamination OR demography OR "disease eradication" OR "disease hotspot*" OR "disease outbreak*" OR "infectious disease transmission*" OR "disease vector*" OR drowning OR emergenc* OR "endemic disease*" OR "environmental exposure" OR "environmental medicine" OR "environmental microbiology" OR "environmental monitoring" OR "environmental pollution" OR "environmental restoration and remediation" OR epidemic* OR "epidemiologic measurement*" OR "epidemiologic method*" OR epidemiology OR "food contamination" OR "food microbiology" OR "food quality" OR "food safety" OR "hand hygiene" OR "health transition" OR hygiene OR "infectious disease incubation period" OR "infectious disease transmission" OR "light pollution" OR "mass drug administration" OR "mass screening*" OR "medical countermeasure*" OR noise OR "nutrition assessment*" OR "nutrition survey*" OR "nutritive value*" OR "occupational medicine" OR "petroleum pollution" OR pharmacovigilance OR "population surveillance" OR "preventive medicine" OR "preventive psychiatry" OR "primary prevention" OR "public health practice*" OR "quaternary prevention" OR "risk assessment" OR "secondary prevention" OR "soil microbiology" OR "spacetime clustering" OR "tertiary prevention" OR "trafficrelated pollution" OR "waste product*" OR "water microbiology" OR "water pollution" ) |
| S17 | (MH "Health Promotion+") |
| S16 | TI ( "preventive health service*" OR "diagnostic service*" OR "diagnostic screening program*" OR "mass screening*" OR "mobile health unit*" OR "early educational intervention" OR "early medical intervention" OR "health education" OR "consumer health information" OR "dental health education" OR "health fair*" OR "health promotion" OR "smoking prevention" OR "immunization program*" OR "mass vaccination*" OR "primary prevention" OR immunization OR "preexposure prophylaxis" OR "quaternary prevention" OR "secondary prevention" OR "tertiary prevention" OR "vaccination coverage" ) OR AB ( "preventive health service*" OR "diagnostic service*" OR "diagnostic screening program*" OR "mass screening*" OR "mobile health unit*" OR "early educational intervention" OR "early medical intervention" OR "health education" OR "consumer health information" OR "dental health education" OR "health fair*" OR "health promotion" OR "smoking prevention" OR "immunization program*" OR "mass vaccination*" OR "primary prevention" OR immunization OR "preexposure prophylaxis" OR "quaternary prevention" OR "secondary prevention" OR "tertiary prevention" OR "vaccination coverage" ) |
| S15 | (MH "Preventive Health Care+") |
| S14 | MH Public Health OR MH Accidental Falls OR MH Safety OR MH Accidents OR MH Accidents, Aviation OR MH Accidents, Home OR MH Accidents, Occupational OR MH Accidents, Traffic OR MH Air Microbiology OR MH Air Pollution OR MH Body Burden OR MH Chemometrics OR MH Infection Control OR MH Decontamination, Hazardous Materials OR MH Demography OR MH Disease Eradication OR MH Disease Hotspot OR MH Disease Outbreaks OR MH Disease Transmission, Horizontal OR MH Disease Vectors OR MH Drowning OR MH Emergencies OR MH Environmental Exposure OR MH Medicine, Environmental OR MH Environmental Microbiology OR MH Environmental Monitoring OR MH Environmental Pollution OR MH Disease Outbreaks OR MH Epidemiology OR MH Food Contamination OR MH Food Microbiology OR MH Food Quality OR MH Food Safety OR MH Handwashing OR MH Epidemiological Transition OR MH Hygiene OR MH Disease Transmission, Vertical OR MH Mass Drug Administration OR MH Medical Countermeasures OR MH Noise OR MH Nutritional Assessment OR MH Nutritive Value OR MH Occupational Medicine OR MH Pharmacovigilance OR MH Population Surveillance OR MH Preventive Health Care OR MH Risk Assessment OR MH Traffic Pollution OR MH Waste Products OR MH Water Microbiology OR MH Water Pollution |
| S13 | S1 OR S2 OR S3 OR S4 OR S5 OR S6 OR S7 OR S8 OR S9 OR S10 OR S11 OR S12 |
| S12 | TI ((child* OR maternal ) N3 health* ) |
| S11 | TI (communit* N3 (based OR care OR health* OR network* OR nursing OR outreach OR participat* OR service* OR support )) |
| S10 | TI (primary N3 (care OR health* )) |
| S9 | TI ("general practi*" OR "family practi*" ) |
| S8 | (MH "Physicians, Family") |
| S7 | (MH "Maternal-Child Care") |
| S6 | (MH "Child Health Services") |
| S5 | (MH "Community Health Nursing+") |
| S4 | (MH "Primary Nursing") |
| S3 | MH "Community Health Services" OR MH "Community Mental Health Services" OR MH "Community Mental Health Nursing" OR MH "Community Networks" OR MH "Consumer Participation" OR MH "Correctional Health Services" OR MH "Counseling" OR MH "Family Planning" OR MH "Family Services" OR MH "Foster Home Care" OR MH "Home Health Care+" OR MH "Maternal Health Services+" OR MH "Occupational Health Services" OR MH "Rehabilitation, Community-Based" OR MH "Senior Centers" |
| S2 | (MH "Family Practice") |
| S1 | (MH "Primary Health Care") |
