# Supplementary material for: Population-oriented health promotion and disease prevention interventions in primary healthcare: a scoping review of reviews
Source: BMC Prim Care. 2026 Apr 22;27:154. doi: 10.1186/s12875-026-03337-y (PMC13104254; doi:10.1186/s12875-026-03337-y)
Supplement: Supplementary file 4 — Supplementary Material 4. [file 12875_2026_3337_MOESM4_ESM.docx]

Additional file 3. Explanation of all data in Additional file 4

Review level:

**Study ID:** Last name of first author and publication year of the review article.

**Title**: The title of the review article.

**Aim of review**: The aim of the review article.

**Type of review**: The type of review article

**Databases sourced and searched in review:** The databases that each review have searched to find their results.

**Time range of database search in review:** The range of years for which the review has limited their database search.

**Target area number:** Unique number (1-11) for each Target area

**Target area**: The health area that the article focuses on.

**Number of articles from the review relevant to the target health area**: The number of articles included from the review article within the target health area.

Article level:

**First author**: Last name of the first author of the article.

**Publication year**: Publication year of the article.

**Study design**: Study design as reported in the article.

**Country:** The country/ies where the intervention/s in the articles were implemented.

**Target group 1**: Population group targeted by the intervention (see different categories of target groups below). In some cases, multiple categories are combined for a specific target group, in which case we have used a plus sign to illustrate that all categories are included in the target group.

**Target group 2/3/4**: The second/third/fourth target group, in cases where the intervention targeted more than one population group.

**Number of target groups:** Number of groups that the intervention target.

**Types of target groups:**

- **Children ≤5**: Children up to five years old.
- **Children>5:** Children between six and 17 years old.
- **Adults**: Individuals aged 18–64.
- **Older adults**: Individuals aged 65 and older.
- **General population**: When the intervention is directed towards the general population rather than specific population groups, e.g. primary care patients.
- **Pregnant/postpartum:** Pregnant women or women during the postpartum period.
- **Women**: Women as reported in study.
- **Men**: Men as reported in study.
- **Caregivers**: Individuals responsible for or contributing to someone else's health, e.g., parents, families, caregivers, or relatives.
- **Minority group**: Minority groups in society, such as African American and indigenous groups.
- **Migrants**: All types of migrants or refugees.
- **Risk group:** In a risk group to get a disease, see different types of risk groups below.

**Types of risk groups:**

- **Risk CVD**: Risk group for cardiovascular diseases.
- **Risk cancer**: Risk group for various forms of cancer.
- **Risk tobacco**: Risk group for various health problems because of tobacco use.
- **Risk alcohol**: Risk group for various health problems because of alcohol use.
- **Risk SES**: Risk group for various health problems because of low socioeconomic status.
- **Risk** **mental health**: Risk group for mental health diagnosis.
- **Risk overweight**: Risk group for various health problems because of overweight

**Description of intervention**: Description of the article’s intervention from review (see categorisation of intervention components below).

**Number of intervention components:** Number of intervention components, see categorisation below.

**Types of intervention components:**

- **Screening**: Screening/ testing for early disease detection; assessment of health status for potential follow-up/referral
- **Physical activity**: Physical activity e.g., walking groups or exercise programs.
- **Counselling/education**: Counselling such as brief advice, motivational interviewing, therapy, etc. Education such as group/ individual educational lectures/ materials etc. Information, such as flyers, brochures or posters with educational messages.
- **Practical support**: Practical support aimed at overcoming barriers to health-promoting interventions, e.g., transportation to/from screening or changes in one’s local environment/home to promote healthy behaviours or reduce risk of accidents.
- **Reminder**: Reminder for screening/health check or other health-promoting activity.
- **Motivator:** Intervention using an inspirational person to influence or motivate the target group towards better health, or a person with personal experience of the targeted health situation/condition providing support.
- **Media**: Use of media or marketing to disseminate health-related messages to the target group.
- **Skills Training**: Teaching practical skills to prevent disease and/or improve health, e.g., learning to perform self-breast exams for breast cancer, to cook healthy recipes or mental coping skills to avoid psychological distress.
- **Vaccination**: Providing vaccination.
- **Mobilisation**: Mobilising community groups in different ways to encourage desired health promoting or disease prevention activities, e.g., screening.
- **Social activity**: Social/cultural activities to enhance a health message or counteract social isolation, e.g., communal dinners, theatres or exhibitions.
- **Referral/invitation**: Referral/invitation/linkage to a health-promoting activity or examination. Could be a formal invitation or referral directly from doctor, but also less direct linkage such as provision of community resource lists.
- **Incentive**: Some form of incentive or gift to increase motivation to engage in a health-promoting activity, e.g. a voucher, an oral health kit or pedometers.
- **Pharmacology/nutrition:** Pharmacological or nutritional support e.g. distribution of nicotine replacement therapy, nutritional supplements, contraceptive pills, etc.

**Actor/s involved**: The actors involved in delivering the intervention to the target group.

**Different types of actors**: Number of different types of actors involved in delivering the intervention (see types below).

**Types of actors:**

- **CHW**: Different types of Community Health Workers. Individuals working closely with the population, often living in the same area, with connection to primary healthcare. Can go under different names and have varying length of education e.g. Promotoras, Lady health workers, Lay health advisors, Aganwadi Workers, and Volunteer health worker.
- **GP**: General practitioner or equivalent, physicians working in primary healthcare.
- **Specialist MD**: Specialist medical doctors working in primary healthcare, e.g. Pediatricians.
- **Nurse**: Nurses or specialised nurses working in primary care.
- **Midwife:** Midwife working in primary healthcare.
- **Health worker:** Health workers within primary healthcare whose roles were not specified in the review article.
- **Other professions**: Professionals in primary healthcare who are neither physicians nor nurses (e.g., psychologists or physical therapists).
- **Project Staff**: Individuals specifically employed in the research project to test/evaluate the intervention e.g. researcher or assistant.
- **Pharmacy staff**: Pharmacist and other staff working at the pharmacy.
- **Counsellor**: Counsellors, therapists, coaches, health educators etc.
- **Dental staff**: Any dental professional within general dentistry.
- **Supporting position**: Individuals in a supportive role, e.g., mentors, peers, or facilitators.
- **Other**: Other actors who do not fit into any of the above categories, such as healthcare assistants or exercise specialists.

**Setting**: The setting where the intervention was delivered (see categories below).

**Different types of settings**: Number of different types of settings.

**Types of settings:**

- **Primary healthcare clinic:** Primary healthcare settings such as a health centre or general practitioner clinic.
- **Community**: In a public space in the community, e.g., a park or cultural centre.
- **Home**: In the participant's home.
- **Perinatal/Child healthcare clinic:** Maternal and child health services that cover health care from pregnancy to newborn stage.
- **Sexual healthcare clinic:** Sexual health and family planning clinics that cover health care related to sexual transmitted diseases, contraception, etc.
- **School:** School health services or school nursing services.
- **Pharmacy:** Pharmacy.
- **Dentist:** Primary dental health services, not specialist dentistry.
- **Other:** When the setting does not fit in any of the above categories, e.g. immunisation clinic. Or when the information is too vague to surely know which category to place in, e.g. “clinic”.

**How the target group was offered the intervention**: How the target group was invited to participate in the intervention, see categories below.

**Types of invitation/recruitment methods:**

- **Recruited in clinic**: Participants are recruited in a clinic by a healthcare professional or research staff. Recruitment could happen before, during or after examination in a waiting room, reception, or examination room.
- **Letter/phone call**: Participants are recruited through invitation letter/mail or phone call.
- **Registers/surveys/medical records/screening:** When there is no information on how participants came into contact with the intervention, but there is information on how potential participants were identified. E.g. “through medical records/ registers” or “through screening”.
- **Advertisement:** Participants are recruited through advertisements, such as newspaper announcements, radio broadcasting, posters, or brochures.
- **Community/home:** Participants are recruited through outreach activities in the community or through home visits.
- **Other settings (schools, pharmacy):** Participants are recruited opportunistically in other settings such as schools or pharmacies.
- **Other:** When the participant is recruited in other ways than those listed above, e.g. online. Or when the information is too vague to surely know which category to place in.

**Sample size**: The total number of participants in both the intervention and control groups

**Sample size groups:**

- **<100:** Less than 100 participants
- **100-999:** Between 100 and 999 participants
- **1,000-10,000:** Between 1 000 and 10 000 participants
- **>10,000:** More than 10 000 participants
- **Missing:** The review had no information on the number of participants
